# SMMTM: Motor imagery EEG decoding algorithm using a hybrid multi-branch separable convolutional self-attention temporal convolutional network

**DOI:** 10.1371/journal.pone.0333805

**Published:** 2025-10-23

**Authors:** DianGuo Cao, ZhenYuan Yu, Jinqiang Wang, Yuqiang Wu

**Affiliations:** The College of Engineering, Qufu Normal University, Rizhao, Shandong, China; University of Reggio Calabria: Universita degli Studi Mediterranea di Reggio Calabria, ITALY

## Abstract

Motor imagery (MI) is a brain-computer interface (BCI) technology with the potential to change human life in the future. MI signals have been widely applied in various BCI applications, including neurorehabilitation, smart home control, and prosthetic control. However, the limited accuracy of MI signals decoding remains a significant barrier to the broader growth of the BCI applications. In this study, we propose the SMMTM model, which combines spatiotemporal convolution (SC), multi-branch separable convolution (MSC), multi-head self-attention (MSA), temporal convolution network (TCN), and multimodal feature fusion (MFF). Specifically, we use the SC module to capture both temporal and spatial features. We design a MSC to capture temporal features at multiple scales. In addition, MSA is designed to extract valuable global features with long-term dependence. The TCN is employed to capture higher-level temporal features. The MFF consists of feature fusion and decision fusion, using the features output from the SMMTM to improve robustness. The SMMTM was evaluated on the public benchmark BCI Comparison IV 2a and 2b datasets, the results showed that the within-subject classification accuracies for the datasets were 84.96% and 89.26% respectively, with kappa values of 0.797 and 0.756. The cross-subject classification accuracy for the 2a dataset was 69.21%, with a kappa value of 0.584. These results indicate that the SMMTM significantly enhances decoding performance, providing a strong foundation for advancing practical BCI implementations.

## Introduction

BCI is a technology that enables direct communication between the brain and external machines [1], allowing computers to interpret brain signals and utilize them to control external devices directly. Consequently, BCI technology holds the potential to transform human life. Currently, BCI have been widely used in human-computer interaction, sports rehabilitation, and disease treatment [2–4]. The main BCI paradigms include steady-state visual evoked potential (SSVEP), P300, and MI [5]. Among these, MI technology is one of the key research focuses in BCI, with MI-based systems already capable of controlling devices such as electric wheelchairs, cursors, and exoskeletons using EEG signals [6–8]. Therefore, MI technology can be applied across various industries, significantly enhancing people’s lives. However, the practical applications of MI remain constrained by the low accuracy of signal decoding, limiting its broader implementation.

Currently, two main types of decoding methods exist for MI-EEG signals: machine learning (ML) and deep learning (DL) [9]. ML has two parts: feature extraction and classifier design. Generally, before extracting EEG features, it is essential to remove signals and artifacts unrelated to the EEG, such as electromyography (EMG), electrooculography (EOG), and electrocardiography (ECG) [10]. Subsequently, temporal and spatial features are extracted from the processed signals to decode the EEG data [11]. After feature extraction traditional ML algorithms, such as support vector machine (SVM) and k-nearest neighbour (KNN) methods, are used to classify MI [12,13]. However, removing artifacts and extracting of features require rich prior knowledge, and the performance of ML algorithms is dependent on the feature selection [14].

DL can automatically extract specific features from raw EEG signals without the need for manually designed features [15]. Over the past six years, utilizing DL for MI classification has increased significantly [16]. Several architectures have been proposed for decoding MI signals, including recurrent neural network (RNN) [17], deep belief network (DBF)[18], and convolutional neural network (CNN) [19]. Heilmeyer FA et al. [20] studied different deep CNNs that can achieve comparable accuracies to those of traditional methods in EEG decoding tasks. Compact EEGNet [21] extracts both the temporal and spatial features of EEG signals by using different convolution kernel shapes, while generalizing to multiple EEG datasets and achieving relatively good performance. The temporal convolutional network (TCN) based on CNN was proposed for time series modelling [22], which can exponentially expand the size of the receptive field with a linear increase in the number of parameters. EEG-TCNet [23] uses EEGNet and TCN to build a high-performance network, and inputs the temporal feature outcomes of EEGNet into TCN to acquire high-level temporal features. However, using a single convolution and a convolution kernel cannot efficiently extract high-level features on multiple scales. MTFB-CNN [24] constructs a parallel convolution to acquire high-level features in time-frequency domain. Incep-EEGNet [25] uses an inception-based network model to capture multi-scale features to decode the original signal. In recent research, MSA [26], can calculate multiple valuable temporal sequence features, has been used for decoding EEG signals. ATCNet [27] builds a network model and uses MSA to highlight the most important information in EEG signals, achieving high classification accuracy. AE-FBCSP combines autoencoder-based feature compression with transfer learning to significantly improve cross- and within-subject motor imagery EEG decoding performance across multiple classification tasks [28]. Few-shot transfer learning approaches, have shown promise in improving MI decoding performance and generalization across tasks [29].

Currently, deep learning (DL) methods for decoding two-class motor imagery (MI) tasks have become relatively mature and show potential for further practical applications [30]. However, as the number of classification tasks increases, decoding performance remains limited, and further exploration is required for four-class MI tasks [31].

In this study, we propose the SMMTM model, which extracts the primary spatiotemporal features from EEG signals using SC serial convolution. However, this overlooks a significant amount of valuable information from the intermediate layers of the neural network. To address this issue, we designed an MSC parallel structure to capture multi-scale temporal features. Additionally, in order to overcome the limitation of CNNs in capturing long-term dependencies from time-series data, we introduced the TCN to extract high-level temporal features. Furthermore, through using MSA, we were able to extract more valuable global information from the MI data. To address the issues of weak feature stability and difficulties in extracting hidden information, we proposed a multimodal feature fusion (MFF). Through feature fusion, the multi-scale features extracted by the MSC and the global features extracted by the MSA are fused along the depth dimension to obtain hybrid features, enhancing the representational capacity of the features. Additionally, decision fusion combines the output results of the MSC and TCN to improve the robustness of the final output. Ultimately, SMMTM achieves high classification accuracy in four-class tasks and demonstrates a certain degree of generalization capability. The performance of the SMMTM model was evaluated on the public available BCI-2a and BCI-2b datasets. The main contributions in this paper are as follows:

1. We propose SMMTM, a high-performance MI-EEG decoding model that utilizes SC to extract primary spatiotemporal features, MSC to capture multi-scale temporal features, MSA to extract global temporal features, TCN to capture high-level temporal features, and MFF to enhance the robustness and generalization of the results.
2. To address the issue of insufficient feature extraction across different frequency bands, filters of varying lengths were designed for each branch, enabling the multi-branch structure to capture multi-scale temporal features. Results from ablation experiments demonstrate that the MSC significantly improves classification accuracy of MI. And the effectiveness of MSC in extracting multi-scale temporal features is demonstrated through weight visualization methods, while the rationality of the SMMTM model is validated using t-SNE visualization techniques.
3. The proposed model achieves excellent results on the publicly available datasets BCI Competition IV-2a (BCI-2a) and BCI Competition IV-2b (BCI-2b), outperforming most of the existing models in terms of classification accuracy.

## Prosed method

### Overall structure of SMMTM

**Fig 1:**
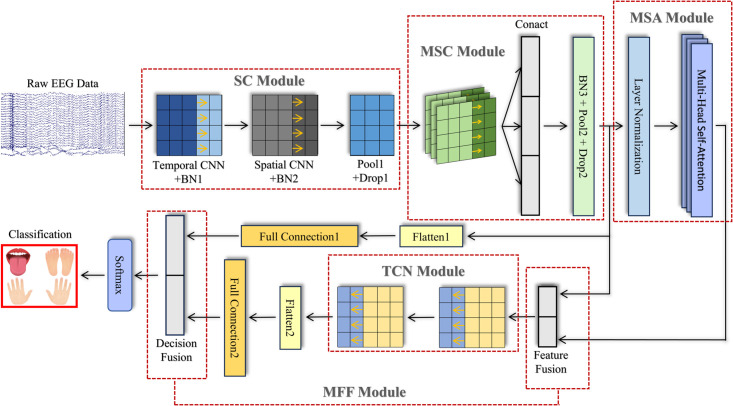
The structure of SMMTM.

In this paper, we propose the SMMTM model, which consists of five modules. The structure of the SMMTM model is illustrated in Fig 1, comprising the SC module, MSC module, MSA module, TCN module, and MFF module.

Initially, spatiotemporal features are extracted using a SC, which consists of serial temporal and spatial convolutions. Subsequently, the MSC module, which is composed of multi-branch separable convolutions, captures temporal features on multiple scales. The output features are then passed to the MSA to highlight valuable information within the temporal sequence. Through feature fusion, the features obtained from MSC and MSA are merged along the depth dimension, and subsequently passed to the TCN to extract high-level temporal features. The results generated by MSC and TCN are then passed to the decision fusion module to enhance classification accuracy and robustness. Finally, the classification results of the SMMTM model are produced using a SoftMax function.

### Spatiotemporal convolution module

EEG signals exhibit distinct spatiotemporal characteristics. The temporal dynamics and spatial distribution of the signals are critical for MI classification tasks tasks [32–34]. Therefore, the temporal convolution in the spatiotemporal convolution (SC) module is used to extract the dynamic temporal features of EEG signals, and the spatial convolution is used to extract the spatial distribution features of electrodes. The combination of temporal and spatial convolutions has been proven essential for the efficient classification of EEG signals in multiple studies [21,27,35]. The SC module consists of serial temporal and spatial convolutions. Initially, temporal convolution is applied to extract primary temporal features, followed by spatial convolution to capture primary spatial features. This dual-feature extraction is crucial for EEG-based tasks, as it allows the model to simultaneously process temporal series over time and spatial relationships across different EEG channels. By integrating temporal and spatial information, SC enhances the model’s ability to understand complex brain activities.

The core of the SC consists of two convolutional layers. The first layer is the temporal CNN. We utilize *F*_1_ = 32 convolution kernels with a size of (1, 32) temporal filters to obtain time features from MI-EEG signals. The second layer is the spatial CNN, which uses depthwise convolution with *F*_2_ convolution kernel of size (C, 1) spatial filters to capture spatial features. C represents the number of EEG channels, with 22 for BCI-2a and 3 for BCI-2b. Preliminary experiments with additional temporal CNN layers showed minimal improvement in representation but led to overfitting and reduced generalization due to the limited sample size of the datasets. Therefore, using a single layer for both temporal and spatial convolutions strikes an optimal balance between feature extraction and model generalization. The dimension of the output feature from the SC module is *F*_2_ = $D\;\times \;F_{1}$, where D is typically set to 2, representing the number of filters linked to the current and previous layers. Following the spatial convolution, an average pooling layer is applied with a kernel size of (1, 8) and a stride of (1, 8). The pooling operation reduces the signal sampling rate to 32 Hz.

### Multi-branch separable convolution module

**Fig 2:**
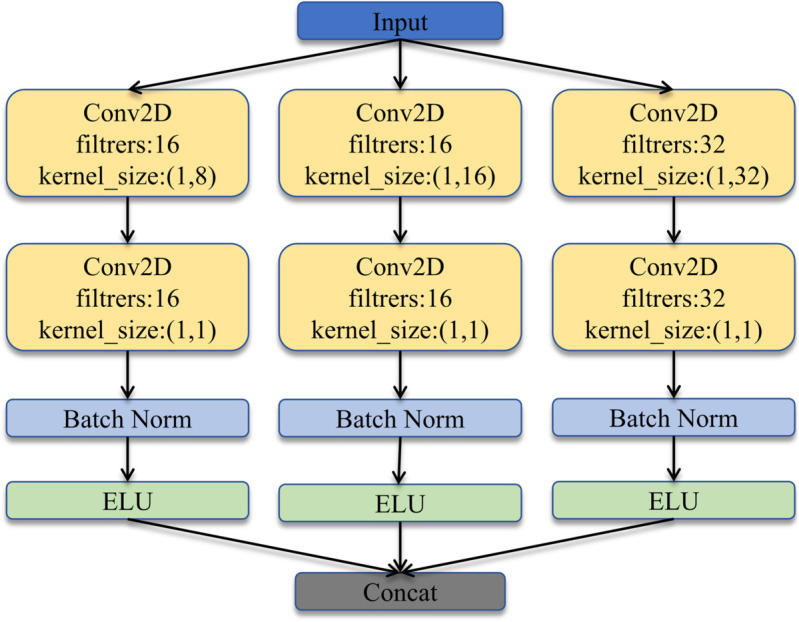
Structure of multi-branch separable convolution.

EEG signals have multi-band characteristics, to capture the differences in EEG signals across different frequency bands, the multi-branch separable convolution MSC was designed. This approach enables the model to take into account both low-frequency and high-frequency components simultaneously, effectively enhancing the model’s ability to understand the signals. The MSC module builds upon the Inception architecture which has proven effectiveness in multi-scale feature extraction [25]. The MSC module consists of three branches, as visualized in Fig 2.

The selection of three branches is motivated by the need to cover short-, medium-, and long-range temporal dependencies. Each branch consists of two convolution layers. The first convolutional layers in the three branches use kernel sizes of (1, 8), (1, 16), and (1, 32), respectively, to capture temporal features at multiple scales that are characteristic of EEG signals. Each branch employs depthwise separable convolutions, where the second layer uses a kernel size of (1, 1) for channel-wise integration, enabling efficient feature extraction with fewer parameters. In the MSC module, the number of filters in the first and second convolution layer is set to *F*_2_/4 for both the first and second branches. In the third branch of the MSC module, the number of filters in the first and second convolution layer is *F*_2_/2. Batch normalization (BN) is used after the second convolution layer to enhance the generalization capability of the model. The BN layer is followed by a nonlinear exponential unit (ELU) activation function. The feature outputs from the three branches are then concatenated in the depth dimension.

### Multi-head self-attention module

**Fig 3:**
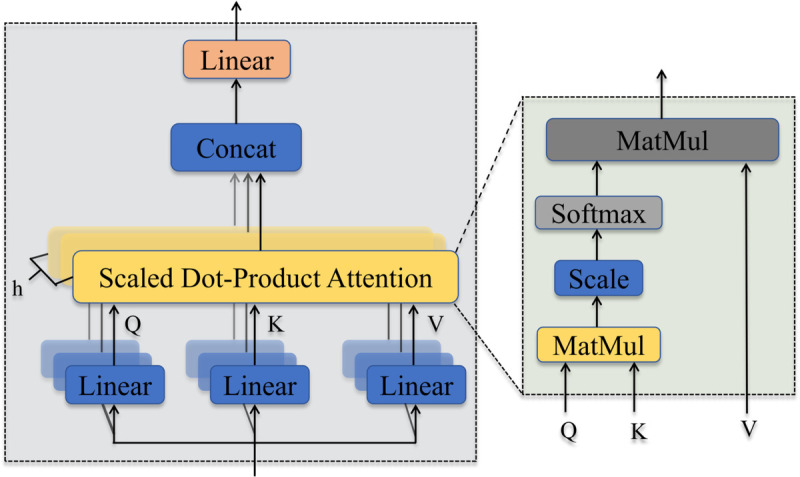
Structure of multi-head self-attention.

The attention mechanism can mimic the behavior of the human brain by selectively focusing on important elements while ignoring others. Integrating the attention with deep learning model helps automatically focus on the most important parts of the input data through learning. MSA [36] is a technique widely used in computer vision and natural language processing [37], capable of computting multiple global time-dependent features in parallel to enhance model accuracy. Therefore, we utilize MSA to further extract critical information from the global temporal sequence. MSA takes each element in the same sequence as input, computes and aggregates attention weights between the elements to obtain the representation of each element. The structure of the MSA is shown in Fig 3.

Calculating multi-head attention, for an input sequence $Y=(y_{1},y_{2},...,y_{m})$, typically involves three main steps:

1. Linear transformation: The input sequence *Y* undergoes three sets of learnable linear transformations to obtain the three vectors *Q* (query), *K* (key), and *V* (value) of the sequence. These formulas can be calculated as follows:

$$Q=Y\cdot W_{Q},$$ (1)

$$K=Y\cdot W_{K},$$ (2)

$$V=Y\cdot W_{V}.$$ (3)

These linear transformations allow the model to learn the relationships and dependencies among various elements in the input sequence. These elements are then used in the subsequent attention calculation.

2. Attention calculation: Calculate the attention distribution for each element in the sequence to obtain an attention vector of length *m*. The formula is as follows:

$$ATscores\;=\;Softmax(\frac{QK^{T}}{\sqrt{d_{h}}}),\;Attention(Q,K,V)\;=\;Dropout(ATscores)V,$$ (4)

where $\sqrt{d_{h}}$ is the square root of the *K* vector dimension. A dropout with a drop rate of 0.3 is used to prevent overfitting.

3. Multi-head attention: The m attention vectors obtained through the multi-head attention are fused, and the head attention vectors are concatenate to form a representation of length m, which is calculated as follows:

$$head_{i}\;=\;Attention(Q_{i},K_{i},V_{i}),\;Z_{m}\;=\;Concat(head_{1},\ldots ,head_{m})W^{o},\;$$ (5)

where *m* = 8 is the number of attention heads and $Q_{i},K_{i},V_{i}$ is the i-th attention head. The MSA builds upon single-head attention. The embedding sequence is transformed through head projection to acquire *Q*,*K*,*V*. Contact represents a concatenation operation, *W*^o^ is an adjustable weight matrix utilized to map concatenated attention to the final representation. MSA simultaneously outputs self-attention projects, allowing it to consider information simultaneously from distinct subspaces at various locations.

### Temporal convolutional network module

**Fig 4:**
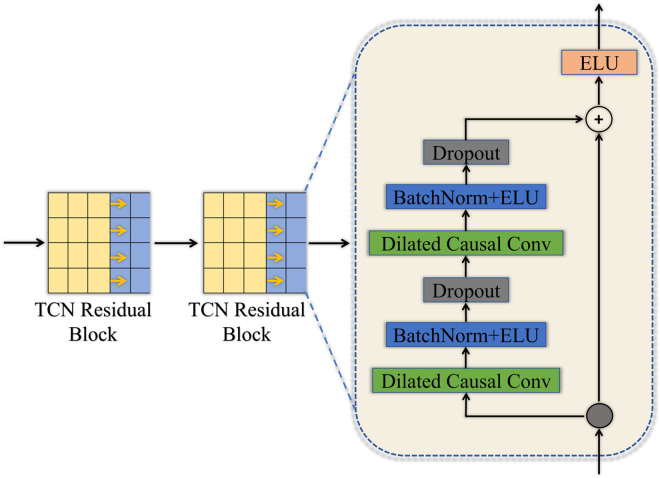
Structure of the TCN consisting of two residual blocks.

TCN have gained significant attention in recent years for the effectiveness in processing sequential data. TCN have been widely applied in various fields, such as time-series forecasting, natural language processing, and EEG signal decoding, due to the ability to capture long-range dependencies while maintaining stability during training. TCN does not require explicitly maintaining the state of the sequence data, which leads results in more efficient computation and the ability to capture longer temporal dependencies. The TCN module has the same architecture as described in [23]. The structure of the TCN is shown in Fig 4. TCN comprises multiple residual blocks, each consisting of two dilated causal convolutions. BN [38], ELU activation and dropout are applied after each layer, as shown in Fig 4.

Causal convolution [39] constrains the convolution kernel to the current and previous time steps to prevent the SMMTM from learning future data. Dilated convolution [40] enhances the receptive field of the SMMTM without requiring additional convolutional layers.

Therefore, dilated causal convolution enables the model to capture information from extended time series. Point convolutions are utilized as residual connections when dilated causal convolution transforms the data into a different dimensionality, which is then input to the residual block. In the SMMTM model, the dimensions of both the input and output residual blocks for the MI-EEG data sequences are $2\;\times \;F_{2}$. Therefore, identity mapping is used for the residual connection.

The dilated causal convolution expansion factor increases exponentially with the number of residual blocks L. For the i-th residual block, the expansion factor is 2^*i*-1^. The receptive field size (RFS) for TCN module is described as follows:

$$RFS=1+2(K_{t}-1)(2^{L}-1).$$ (6)

In the SMMTM, the input sequence length of TCN is 15, the count of residual blocks L is 2. The RFS needs to be larger than the duration of the input sequence to prevent loss of information during the convolutional process. As a result, the kernel size of the convolutional layer is defined as *K*_*t*_ = 4, resulting in an RFS of 19, which is greater than 15.

The output of the TCN module is final element in the sequence, represented by a vector of size *F*_*T*_ = $6\;\times \;F_{2}$. TC blocks from all windows are concatenated and then sent to the FC layer. Fig 1 illustrates this architecture.

### Multimodal feature fusion module

To improve the model’s decoding accuracy and stability, we introduced the MFF module, which consists of feature fusion and decision fusion, as shown in Fig 1. Detailed architectural dimensions and computational complexities are as follows:

Feature fusion combines the output from the SMMTM to capture hidden information and enhance the feature representation. The MSC module outputs features of dimension (*F*_2_,1,*T*//64), where *F*_2_ represents the feature depth and *T*//64 denotes the temporal dimension. Feed the features output by the MSC module into the MSA module. Since the MSA module does not change the dimensions of the input features, the feature dimensions output by the MSA module are (*F*_2_,*T*//64). The features output from the MSC convolution are multi-scale features, and the output of the MSA module is global features. Therefore, the features output from the MSC and the MSA module were concatenated along the depth dimension to obtain a comprehensive fusion feature. Then, the fusion feature is fed into the TCN to extract higher-level time-dependent information. Decision fusion can be used to merge the output multiple classifier results, reducing the uncertainty or error of a single classifier. The prediction results of the TCN and MSC modules are integrated to achieve a more accurate probability. Finally, the probability is inputted into the SoftMax function to obtain the final classification result. The detailed configuration and parameter settings of the proposed model are summarized in Table 1.

**Table 1:** Propose the parameters of the model, where *C* is the number of channels, *T* is the number of sample points, $F_{1}$ is the number of temporal filters, *D* is the depth multiplier, $F_{2}=D\;\times \;F_{1}$, $N_{C}$ is the number of classifications.

Layer	Filters	Kernel size	Output	Activation	Options
Temporal	*F*_1_	(1, 16)	(*F*_1_,*C*,*T*)		padding = same,
CNN					bias = false
BN1			(*F*_1_,*C*,*T*)		
Spatial	$D\;\times \;F_{1}$	(*C*,1)	$\left(D\;\times \;F_{1},C,T\right)$		padding = same,
CNN					bias = false,
					groups = *F*_1_
BN2			$\left(D\;\times \;F_{1},1,T\right)$	ELU	
Pool1		(1, 8)	$\left(D\;\times \;F_{1},1,T//8\right)$		
Drop1			$\left(D\;\times \;F_{1},1,T//8\right)$		p = 0.5
Multi-branch					
separable			$\left(D\;\times \;F_{1},1,T//8\right)$		
convolution					
BN3			$\left(D\;\times \;F_{1},1,T//8\right)$	ELU	
Pool2		(1, 8)	$\left(D\;\times \;F_{1},1,T//64\right)$		
Drop2			$\left(D\;\times \;F_{1},1,T//64\right)$		p = 0.5
Flatten1			$\left(T\;\times \;F_{2}//64\right)$		
FC1			*N*_*C*_		max norm = .25,
					init bias = 0.0
LN			(*F*_2_,*T*//64)		
MSA			(*F*_2_,*T*//64)	ELU	heads = 8,
					p = 0.3,
					norm = .25
Concatenate1			$\left(F_{2}\;\times \;2,T//64\right)$		cat(Drop2, MSA)
TCN	$2\;\times \;F_{2}$	(1,*K*_*t*_)	$\left(6\;\times \;F_{2}\right)$	ELU	p = 0.5,
					bias = false,
					max norm = .5
Flatten2			$\left(6\;\times \;F_{2}\right)$		
FC2			*Nc*		max norm = .25,
					init bias = 0.0
Concatenate2			*N*_*C*_		cat(FC1,FC2)
Softmax			*Nc*		

## Experiments

### Datasets

This section presents the test results obtained from the EEG data of the famous BCI competition dataset, to validate the effectiveness of the SMMTM.

First, the dataset and experimental settings are presented. Then, we describe the results and discuss our findings. In this work, the SMMTM model was evaluated on the BCI Competition IV-2a [41] and 2b [42] datasets, which have been widely used in the research community and are thus considered benchmark datasets in MI-EEG decoding.

**Fig 5:**
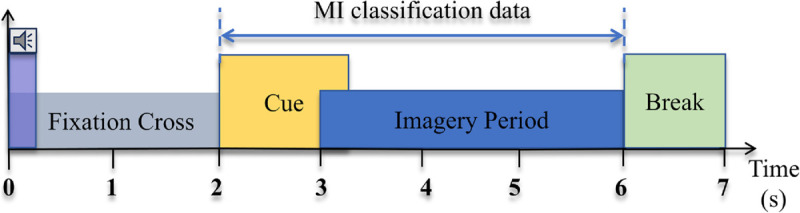
Timing scheme for each trial, with 4 seconds of MI activity.

This section provides a description of the 2a dataset, which includes the EEG data of four MI movements of nine subjects. The data were collected on separate days during two sessions for each subject. Both sessions included training and test sets. The EEG data were recorded at a sampling frequency of 250 Hz using 22 Ag/AgCl electrodes. In each trial, the MI data was collected for 4 seconds, as shown in Fig 5.

The BCI-2b dataset includes MI data recorded from 9 subjects using 3 electrodes, with a sampling rate of 250 Hz. Each subject participated in five sessions, which included MI tasks for both the left and right hands. For each subject, two training sessions were conducted without visual feedback, followed by three training sessions with visual feedback. The first three sessions were used as the training set, and the last two were used as the test set. The lengths of the tests in the experiment were between 3 and 7 seconds.

### Data preprocessing

Data preprocessing is employed to eliminate noise and artifacts. EEG feature extraction plays a significant role in identifying the subject’s imagined movements before classification. In this study, we do not preprocess the raw EEG signals and input MI-EEG signals $X_{i}\in {\mathbb{R}}^{C\;\times \;T}$ into the SMMTM, which included *C* channels and *T* sample points. We applied Z-score normalization to eliminate variability in the EEG signals to enhance training speed and improve model accuracy:

$$x_{0}=\frac{x_{i}-\mu }{\sqrt{\delta^{2}}},$$ (7)

where the standardized output and training/test data are denoted as *x*_0_ and *x*_*i*_, respectively. The average *μ* and variance $\delta^{2}$ of the training data are calculated, and used to standardize the training and test data.

### Experimental details

SMMTM is implemented using the PyTorch 1.12 DL framework in Python 3.7. The training process is performed on an Nvidia GTX 3090 24 GB. We use the Adam optimizer to optimize the model, with the following hyperparameters: learning rate of 0.001, $\beta_{1}=0.9$, $\beta_{2}=0.99$, and weight decay is 0.001. The overall model loss is computed using the cross-entropy function. The calculation formula is as follows:

$$l(x,y)=\sum_{m=1}^{M}\frac{l_{m}}{M},\;l_{m}=-\log\frac{exp(x_{m},y_{m})}{\sum_{m=1}^{N_{C}}exp(x_{m},c)},$$ (8)

*x* represents the output of the model, *y* is the label, *M* corresponds to the batch size.

The classification accuracy and kappa score are employed as evaluation metrics to assess the overall performance of the model:

$$kappa=\frac{p_{a}-p_{e}}{1-p_{e}},\;Acurary=\frac{TP+TN}{TP+TN+FP+FN},$$ (9)

*p*_*a*_ denotes the model classification accuracy, while *p*_*e*_ denotes the expected consistency level of the model, TP and TN respectively represent true positive and true negative, while FP and FN represent false positive and false negative.

The SMMTM was used to conduct cross-subject and within-subject experiments on subjects. Both sets of experiments used 5-fold cross-validation. The training parameters were determined through cross-validation on the validation set. Additionally, an early stopping [43] method was implemented to prevent overfitting. Model training was stopped if there is no significant loss reduction after 300 steps iterations each branch in the EEG decoding network.

## Results

### Ablation study

Ablation experiments were conducted on the BCI-2a to evaluate the impact of each layer of the SMMTM on EEG decoding performance. Table 2 presents the within-subject decoding results on BCI-2a for experiments where each module was individually removed, with the best results highlighted. Modules were removed prior to the training and validation procedures. The training parameters are the same as those described in Within-subject Decoding Experiment. The model’s average accuracy decreased by 5.5% with the removal of the MSC module and by 6.7% with the removal of the TCN module, indicating that effective temporal feature extraction significantly contributes to improving signal decoding accuracy. When the MSA module was removed, the model’s average accuracy drops to 82.7%. Adding feature fusion ahead of the TCN module improves the accuracy of the SMMTM by 2.6%. Using decision fusion after the TCN module enhances the accuracy of the SMMTM by 1.5%. The accuracy of the SMMTM is increased by 3.3% by applying MFF. The test results demonstrate that each module effectively improves EEG decoding accuracy.

**Table 2:** Ablation experiments: Mean accuracy and k-score of deep learning models with various combinations.

Removed block	Accuracy%	K-score
None(SMMTM)	**84.9%**	**0.797**
MSC	79.4%	0.748
TCN	78.2%	0.732
MSA	82.7%	0.764
feature fusion	82.3%	0.757
decision fusion	83.4%	0.762
MFF	81.6%	0.751

### Within-subject decoding experiment

The within-subject decoding performance of the SMMTM was tested on BCI-2a and BCI-2b and compared with that of other excellent algorithms, some of which were based on a reproduction of the original paper. Tables 3 and 4 list the MI decoding accuracy, average accuracy (percentage), k-score, p-value, and std of nine subjects. The best data is highlighted. The results show that the proposed algorithm outperforms other algorithms. The findings illustrate that the model showed good decoding performance in both datasets and achieved higher classification accuracy for poor subjects. In the 2a dataset, topic seven showed the best MI encoding effect, achieving an accuracy rate of 93.06%. In the 2b dataset, topic four showed the best MI encoding effect, achieving an accuracy rate of 96.56%. SMMTM was compared with other algorithms using the Wilcoxon signed-rank test. The results show that SMMTM significantly outperformed most algorithms (*p* < 0.05), and its decoding accuracy was higher than that of algorithms where the difference was not statistically significant.

**Table 3:** Within-subject comparison of decoding performance of state-of-the-art-methods on BCI-2a.

Method	S01	S02	S03	S04	S05	S06	S07	S08	S09	Avg	K-score	Std	p-value
EEGNet* [21]	83.04	58.44	88.13	61.28	71.58	55.03	80.09	77.71	75.98	72.36	0.665	11.0	0.007
ShallowConvNet* [44]	81.94	58.68	88.54	74.31	75.00	59.72	89.93	84.02	79.51	76.85	0.691	10.7	0.008
DeepConvNet [44]	78.24	52.77	84.08	68.17	61.81	63.19	86.52	76.73	83.15	72.74	0.683	11.1	0.008
EEG-TCNet [23]	85.77	65.02	**94.51**	64.91	75.36	61.40	87.36	83.76	78.03	77.35	0.700	11.5	0.010
TSFCNet [45]	**90.28**	62.50	93.40	83.33	75.35	68.06	92.49	**88.19**	87.85	82.38	0.769	11.6	0.213
CNN-TFCSP [46]	85.76	62.50	87.15	76.04	78.82	59.72	92.36	86.46	84.72	79.28	-	-	0.138
MSFNet [47]	83.68	74.15	90.68	76.90	**78.83**	68.04	88.30	79.52	84.16	80.47	0.743	-	0.313
M-FNet [48]	86.81	75.00	91.67	73.61	76.39	61.46	85.76	75.69	87.15	79.28	0.725	-	0.092
SMMTM	88.89	**77.78**	92.01	**87.85**	76.39	**74.66**	**93.06**	84.73	**89.24**	**84.96**	**0.797**	**6.5**	

*Results reproduced based on the original paper.

**Table 4:** Within-subject comparison of decoding performance of state-of-the-art methods on BCI-2b.

Method	S01	S02	S03	S04	S05	S06	S07	S08	S09	Avg	K-score	Std	p-value
EEGNet* [21]	72.56	70.21	85.51	96.94	91.74	77.94	91.13	93.06	88.06	85.24	0.705	9.0	0.007
ShallowConvNet* [44]	71.25	63.93	77.81	96.56	94.06	87.81	87.19	91.56	85.63	83.98	0.679	10.3	0.008
EEG-TCNet* [23]	75.00	70.43	84.38	96.38	**95.25**	78.44	88.31	92.69	84.19	85.01	0.701	8.6	0.213
TSFCNet [45]	76.25	70.00	83.75	**97.50**	92.81	86.56	88.44	92.50	**89.69**	86.39	0.732	8.6	0.315
CapsNet [49]	78.75	55.71	55.00	95.93	83.12	83.43	75.62	91.25	87.18	78.44	-	-	0.031
MSHCNN [50]	**86.80**	77.94	65.97	96.97	93.24	87.88	86.80	82.89	86.80	85.08	0.729	9.2	0.260
BFATCNet [51]	84.20	77.10	84.60	97.40	87.70	85.40	84.10	89.10	82.90	85.80	0.720	8.2	0.537
SMMTM	84.50	**79.72**	**87.83**	96.56	92.50	**88.13**	**92.82**	**94.07**	86.56	**89.26**	**0.746**	**4.9**	

*Results reproduced based on the original paper.

**Fig 6:**
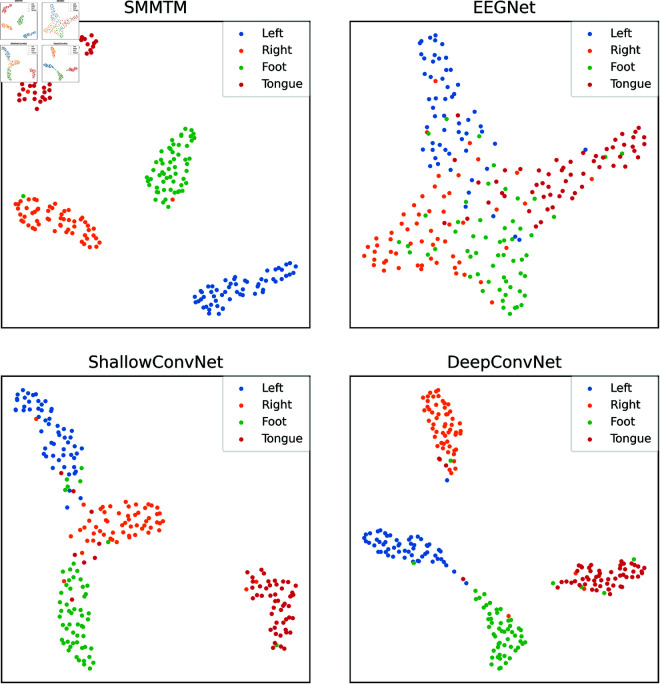
The feature vectors for S07 in BCI-2a are distributed in 2D space using the t-SNE method.

After training with DeepConvNet, ShallowConvNet, EEGNet, and the SMMTM, the feature distribution of subject S07 from the BCI-2a dataset was mapped onto a 2D plane using t-SNE, as illustrated in Fig 6. The visualized feature distribution shows that the features extracted by the SMMTM exhibit more distinct boundaries and well-separated clusters compared to those extracted by ShallowConvNet, DeepConvNet, and EEGNet. This indicates that the SMMTM effectively captures discriminative information, improving the separability of different classes.

**Fig 7:**
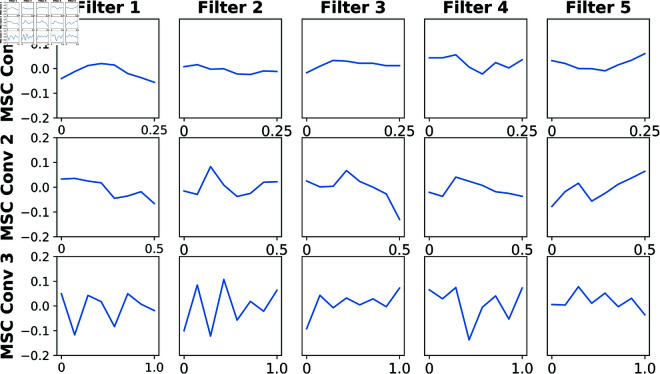
Visualization of the convolutional weights of the MSC for subject 7 in BCI-2a.

The convolutional kernel weights of the MSC are visualized, and the results are shown in Fig 7. The kernels in MSCConvs 1, 2, and 3 have lengths of 8, 16, and 32 samples, corresponding to 0.25, 0.5, and 1 seconds in time, respectively. The frequency bands learned by the MSCConv 1 is high and wide, and the MSCConv 3 is low and narrow. This demonstrates that the MSC can learn multi-scale of band information with different sizes of temporal filters.

**Fig 8:**
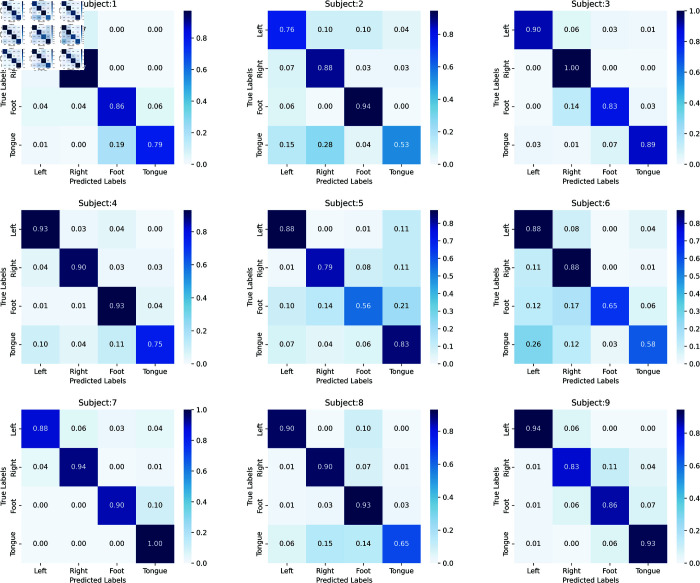
The confusion matrices of the SMMTM on the MI BCI IV-2a dataset.

The test results on BCI-2a indicate that the SMMTM achieves the highest average classification accuracy with the smallest std and the highest k-score, as shown in Table 3. Additionally, the SMMTM outperforms other algorithms on most subjects. The accuracy is 7.61% higher than that of EEG-TCNet, which also uses TCN. 12.6% higher than that of EEGNet, which is a CNN-based model, only captures local information from EEG signals. In contrast, SMMTM enhances the CNN architecture by incorporating MSA and TCN to extract both local and global dependencies, thereby improving decoding performance. 5.68% higher than that of CNN-TFCSP, which also uses self-attention, this demonstrates that attention heads MSA can extract more comprehensive global information. 4.49% higher than that of MSFNet, which also uses multi-branch structure. Fig 8 shows, that we use the confusion matrix to evaluate efficiency of the four topic models. The diagonal line is the overall accuracy for each task. We find that, it worked better when classifying the left and right hands in most case, but not well when classifying the feet and tongues. It was difficult for participant 5 to classify feet and tongue, resulting in low classification accuracy.

The test results of BCI-2b indicate that the SMMTM achieves the highest average classification accuracy with the smallest std and the highest k-score, as shown in Table 4. Additionally, the SMMTM outperforms other algorithms on most subjects. The accuracy is 4.25% higher than that of EEG-TCNet, which also uses TCN; it is 4.02% higher than the accuracy of EEGNet, which also uses SC, and it is 4.18% higher than that of MSHCNN, which also uses multi-branch structure.

### Cross-subject decoding experiment

Table 3 shows the overall accuracy and k-scores for within-subject MI-EEG classification using BCI-2a dataset. Due to the significant within-subject variability in EEG signals, the cross-subject decoding performance of the SMMTM on BCI-2a was tested to measure its generalizability. It was compared with that of other excellent algorithms. The replicated experimental results in Table 5 of this paper were obtained by replicating the network models based on the parameters of the network models in the original paper and training them through the Leave-One-Subject-Out (LOSO) method proposed in this paper. Meanwhile, these models were also experimentally validated in the corresponding public datasets BCI-2a and BCI-2b in the original paper. The calculation results of other network models in Table 5 are the calculation results provided in the original paper, which are directly cited in the table.

**Table 5:** Cross-subject comparison of decoding performance of state-of-the-art methods on BCI-2a.

Method	S01	S02	S03	S04	S05	S06	S07	S08	S09	Avg	K-score	Std	p-value
EEGNet* [21]	63.02	41.34	76.52	52.63	48.61	38.39	70.83	68.26	55.16	57.20	0.429	12.5	0.009
ShallowConvNet* [44]	67.53	43.40	73.82	54.93	43.65	44.03	71.88	74.89	65.52	59.96	0.472	13.3	0.011
EEG-TCNet* [23]	66.08	50.90	73.44	50.94	50.56	49.44	**73.16**	69.72	65.24	61.05	0.481	9.8	0.112
CCNN [52]	62.07	42.44	63.12	52.09	49.96	37.16	62.54	59.32	**69.43**	55.34	-	-	0.009
CRAM [53]	61.02	42.35	73.11	50.43	50.74	51.48	67.26	69.72	66.85	59.22	-	10.1	0.039
G-CRAM [54]	-	-	-	-	-	-	-	-	-	60.11	-	-	0.082
SMMTM	**77.26**	**58.22**	**84.72**	**62.60**	**59.75**	**57.41**	72.83	**81.43**	68.63	**69.21**	**0.584**	**9.7**	

* Results reproduced based on the original paper.

The model was evaluated using the leave one subject out (LOSO) method during cross-subject experiments. This approach involves selecting one subject from the nine subjects employed for testing purposes, while designating the remaining eight subjects as the training set. Table 5 lists the average accuracy (percentage) for each algorithm. The data for the best algorithm is highlighted. The results show that the proposed SMMTM achieves the highest average classification accuracy with the highest k-score, as shown in Table 5. Additionally, the SMMTM outperforms other algorithms on most subjects. The accuracy is 8.16% higher than that of EEG-TCNet, which also uses TCN, and it is 12.01% higher than the accuracy of EEGNet, which also uses SC. The Wilcoxon signed-rank test results indicate that SMMTM significantly outperformed EEGNet (p = 0.009), ShallowConvNet (p = 0.011), CCNN (p = 0.009), and CRAM (p = 0.039). with decoding accuracy was notably higher than that of EEG-TCNet and G-CRAM. These results validate the SMMTM as an effective decoding algorithm for identifying accurately EEG signals.

## Conclusion

This study proposes the SMMTM, a hybrid high performance neural network based on five modules: SC, MSC, MSA, TCN, and MFF. Each module plays a crucial role in improving the model’s performance, as demonstrated by the ablation experiments, which show that the inclusion of each component leads to enhanced decoding accuracy. The model’s interpretability is illustrated through visualization methods, demonstrating the effectiveness of MSC in capturing multi-scale temporal features. The within-subject accuracy of the proposed SMMTM algorithm achieved 84.96% on the BCI-2a dataset and demonstrated superior performance over other state-of-the-art algorithms mentioned in the paper. For cross-subject evaluation on the BCI-2a dataset, the accuracy reached 69.21%, outperforming comparable methods. Additionally, on the BCI-2b dataset, the within-subject results of SMMTM also surpassed the referenced algorithms, further validating its effectiveness across different public datasets.The effectiveness of the proposed algorithm was verified by means of p-value tests on both the BCI-2b and BCI-2a datasets. These results indicate that the SMMTM model is not only capable of capturing intricate features from EEG signals but also robust across subjects, which is critical for real-world applications. The high classification accuracy and robustness of the model suggest that SMMTM could serve as a powerful tool for future BCI applications, potentially improving the efficiency and reliability of BCI systems. In future work, we will attempt to conduct online experimental verification. This will involve real-time implementation and evaluation of the model in practical BCI scenarios, allowing us to assess its real-world performance and make necessary adjustments for optimization.
